# Presenilin Controls CBP Levels in the Adult *Drosophila* Central Nervous System

**DOI:** 10.1371/journal.pone.0014332

**Published:** 2010-12-14

**Authors:** Randy S. Boyles, Kathryn M. Lantz, Steven Poertner, Stephanie J. Georges, Andrew J. Andres

**Affiliations:** School of Life Sciences, University of Nevada-Las Vegas, Las Vegas, Nevada, United States of America; Ludwig-Maximilians-Universität München, Germany

## Abstract

**Background:**

Dominant mutations in both human *Presenilin* (*Psn*) genes have been correlated with the formation of amyloid plaques and development of familial early-onset Alzheimer's disease (AD). However, a definitive mechanism whereby plaque formation causes the pathology of familial and sporadic forms of AD has remained elusive. Recent discoveries of several substrates for Psn protease activity have sparked alternative hypotheses for the pathophysiology underlying AD. CBP (CREB-binding protein) is a haplo-insufficient transcriptional co-activator with histone acetly-transferase (HAT) activity that has been proposed to be a downstream target of Psn signaling. Individuals with altered CBP have cognitive deficits that have been linked to several neurological disorders.

**Methodology/Principal Findings:**

Using a transgenic RNA-interference strategy to selectively silence *CBP*, *Psn*, and *Notch* in adult *Drosophila*, we provide evidence for the first time that Psn is required for normal CBP levels and for maintaining specific global acetylations at lysine 8 of histone 4 (H4K8ac) in the central nervous system (CNS). In addition, flies conditionally compromised for the adult-expression of CBP display an altered geotaxis behavior that may reflect a neurological defect.

**Conclusions/Significance:**

Our data support a model in which Psn regulates CBP levels in the adult fly brain in a manner that is independent of Notch signaling. Although we do not understand the molecular mechanism underlying the association between Psn and CBP, our results underscore the need to learn more about the basic relationship between Psn-regulated substrates and essential functions of the nervous system.

## Introduction

In humans, functional Psn, the catalytic subunit of the γ-secretase complex, is a nine-pass transmembrane protein that contains an aspartyl protease domain. Psn is best characterized for its role in early onset familial forms of Alzheimer's disease (FAD). Patients with FAD often have single gain-of-function mutations in *Psn* that are thought to process amyloid precursor protein (APP) in a manner that releases amyloid (Aβ) fragments into the extracellular matrix. These fragments, usually 40 and 42 amino acids in length, aggregate into the amyloid plaques that are often considered a hallmark of Alzheimer's disease (AD) [1].

Psn is known to cleave 55 substrates [2], the majority of which are single-pass type-I transmembrane receptors that include Notch and APP [3]. Prior to Psn processing, ligand binding usually activates a metalloprotease that processes the extracellular domain (ECD) of the receptor. The truncated receptor then becomes a substrate for Psn, which processes it further to release the intracellular domain (ICD) from the membrane. The ICD normally translocates to the nucleus to co-activate gene transcription. Thus, this regulated intramembrane proteolysis (RIP) activity of Psn [4] couples external signals to changes in the transcription pattern of target genes.

Although extensive investigations have attempted to demonstrate the causative action of amyloid plaques in the manifestation of AD, no clear mechanism has yet been demonstrated for how these plaques cause the overall neurological disease (reviewed in [4]–[6]). Therefore, elucidating the signaling pathways controlled by Psn remains an important area of basic research that could impact future treatments of AD.

One interesting candidate gene that may be regulated through Psn processing encodes CBP [Cyclic-AMP-Response Element-Binding (CREB)-Binding Protein]. CBP is promiscuous and known to interact with over 300 different substrates; 200 of which are categorized as essential in the mouse genome [7], [8]. It is a multi-functional protein that can co-activate transcription factors, bridge enhancer-binding complexes, and acetylate histones (reviewed in [9]). It is often the limiting factor or signal integrator in many important pathways that control nervous system activities. Null mutations in *CBP* are homozygous lethal, but hypomorphs and haploinsufficiencies have been linked to human cognitive disorders including Huntington's disease and Rubenstein Taybi disease, a developmental defect characterized by reduced mental function [10], [11].

The association between Psn and CBP is unlikely to be direct, but mediated through one or more of the several Psn-processed substrates. Previously, we have shown a requirement for Notch in the long-term memory (LTM) of flies [12], and we are testing a molecular model whereby Notch signaling acts as an intermediate between Psn and CBP. In this report, we examine the conflicting relationship between Psn, Notch, and CBP using the conditional genetic reagents of the *Drosophila* model system [13], [14]. Because CBP is needed for many higher order cognitive functions in flies and vertebrates, we are confident that important aspects of the signaling mechanism will be conserved between the two systems. However, although our data suggests that Psn is necessary for maintaining CBP levels and global chromatin acetylation [as monitored at lysine 8 of histone 4 (H4K8ac)] in the adult fly brain, these activities are not dependent on a simple mechanism whereby Psn processes Notch into a transcriptional co-activator that regulates *CBP* expression.

## Results

### Phenotypic-Based Analysis of Transgenic RNAi Lines

Psn is emerging as an important signal integrator in which external cues are transmitted internally through signaling cascades that affect many cellular processes including changes in gene expression. Because Psn contains an aspartyl protease domain, its primary role in this process is thought to occur through proteolytic processing of type I transmembrane receptors. Furthermore, the link between mutations in *Psn* and inherited forms of AD has placed a premium on trying to understand the intricate signaling network mediated by this complex. The powerful genetic tools unique to *Drosophila* are particularly valuable in trying to understand this signaling mechanism because the *Gal4/UAS* binary expression system [15] can be used to over-express and/or silence virtually any gene in the organism in a temporally and spatially specific manner.

We began this analysis by first trying to understand what happens to flies when protein levels of Psn, Notch, and CBP are reduced in the adult nervous system. Our approach was to use an inducible-RNAi strategy that allows these genes to be fully functional during embryonic and larval development, but to silence them in selected adult tissues.

**Figure 1:**
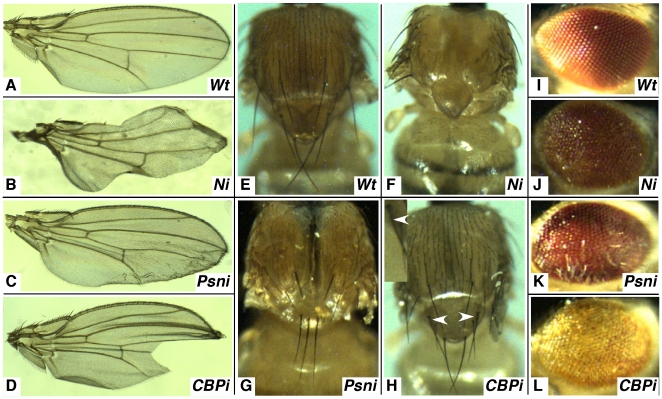
Developmental phenotypes of flies compromised for *Notch*, *Psn* or *CBP* using transgenic RNA*i*. (A) represents a wildtype wing and (B–D) show phenotypes of the *UAS-RNAi* lines crossed to *c96-Gal4*. Silencing either gene with this driver results in irregular or missing wing margins and wrinkling and notching of the wing blade. (E) is a wildtype thorax and (F–H) are representative samples of the *UAS-RNAi* lines crossed to *pnr-Gal4*. Silencing the genes with this driver leads to improper sensory organ development that gives rise to balding and/or supernumerary machrochete on the thorax and notum. (I) is a wildtype eye and (J–L) represent *UAS-RNAi* lines crossed to *GMR-Gal4*, which causes blistered and fused ommatidia, and with the *Psn* knockdowns in particular (K), sporadic elongated sensory hairs.

We have previously described the generation and characterization of several transgenic lines of flies that placed an inverted repeat of *Notch* sequence under control of GAL4 transcription factor binding sites (*UAS elements*). We produced 24 lines and we showed that all could selectively silence the *Notch* gene to some degree. However, we demonstrated that one line, *UAS-Ni-14E* (hereafter referred to as *UAS-Ni*) effectively reduced the accumulation of Notch protein (as judged by western analysis) and eliminated Notch signaling when expressed in target tissues [16]. For example, Notch signaling has been shown by mutant analysis to be required for the proper specification of the wing margin [17]. Thus, when *UAS-Ni* is expressed in that tissue using the *c96-Gal4* driver, the wing margin is dramatically scalloped and almost devoid of trichomes (Fig.1B). Notch is also required for proper patterning of macro- and microcheate sensory bristles on the thorax [18], and when *UAS-Ni* is expressed in that tissue using a *pnr-Gal4* driver, a disruption of bristle pattern and balding is apparent (Fig. 1F). Finally, it has been demonstrated that Notch is needed for R8 photoreceptor specification in the developing eye disc [19], and when it is silenced in the developing eye disc with the *GMR-Gal4* driver, the eye appears disorganized and roughened (Fig. 1J).

Because Psn is known to process Notch into a signaling molecule, we used the same logic to judge the effectiveness of twelve *UAS-Psni* lines generated in our lab or collected from the *Drosophila* community (see Methods). The most efficacious line produced wrinkles and notches in the wing blade (Fig. 1C), bristle fusion and elimination on the thorax (Fig. 1G), and roughening of the eye (Fig. 1K).

Finally, because we expected Psn, Notch, and CBP to be involved in a related molecular pathway (see below), we examined the phenotypes produced when *CBP* was silenced with RNAi. We obtained a *UAS-CBPi* line that was shown to be an effective reagent for silencing the endogenous *CBP* gene [20]. Interestingly, when we expressed the *UAS-CBPi* construct in the same three tissues using the same three drivers, we observed similar phenotypes to those seen when *Notch* and *Psn* were silenced: wrinkles and notches in the wing blade (Fig. 1D), machrocheate fusion (Fig. 1H, arrowheads and insert), and a roughened appearance of the eye (Fig. 1L).

### Presenilin Regulates CBP in a Notch-Independent Manner

We and others have demonstrated that Notch is needed for long-term memory (LTM) in flies and mice [12], [21], [22]. Others have also shown that the CREB transcription factor is important in the same process in the same organisms [23], [24], and that a key co-activator of CREB-mediated transcription is CBP. CBP is also required for LTM [25], and has been hypothesized to be a target gene for Notch signaling [13]. Thus, we began testing a model in which the Psn-dependent release of the Notch ICD transcriptionally controls the expression level of *CBP* in the adult nervous system of flies.

**Figure 2:**
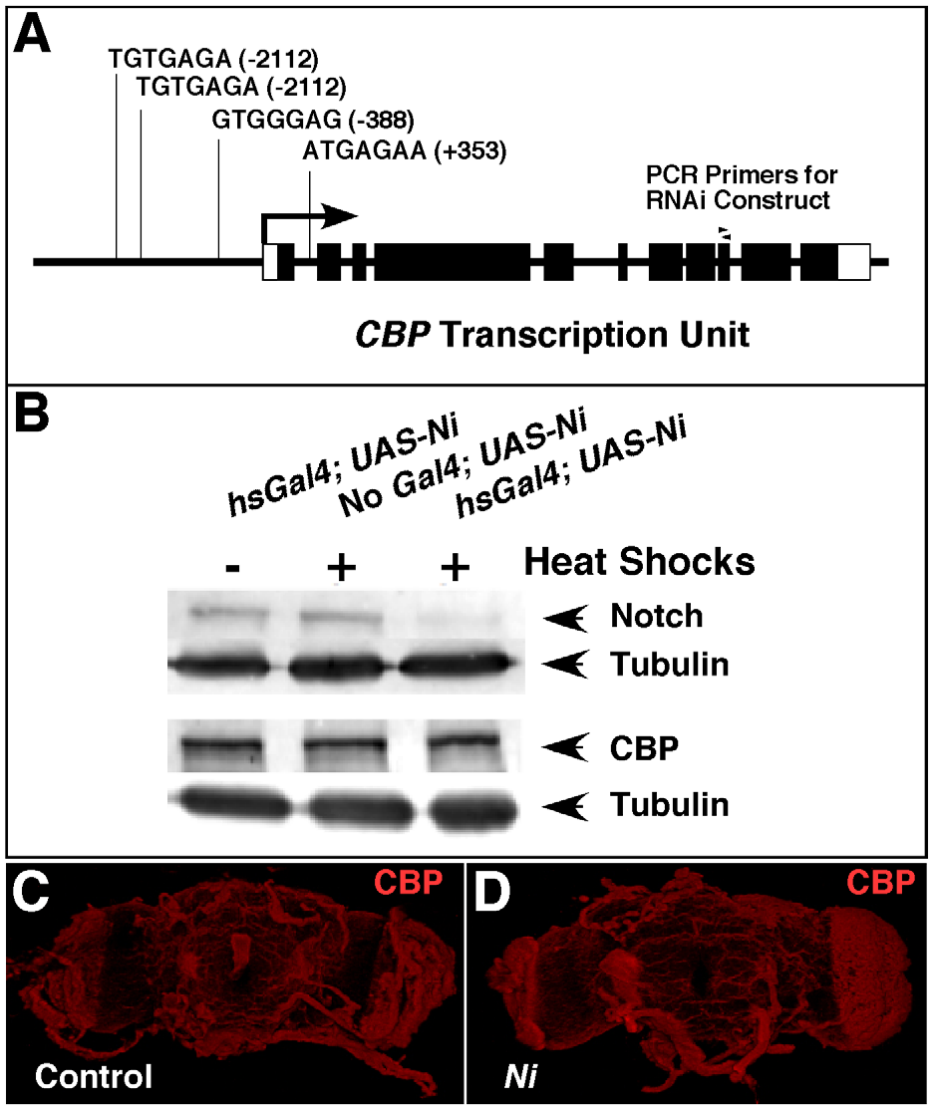
Notch is not required for *CBP* expression. (A) shows the location of four putative binding sites for Su(H) near the transcription unit of *CBP*. Boxes indicate exons and filled regions represent sequences that are translated. The position of each element is indicated relative to the start of transcription (+1, arrow). (B) is a western analysis of Notch and CBP protein levels under conditions in which *Notch* is silenced with RNAi by applying multiple heat shocks (see Materials and Methods). Two blots were made from each protein sample. One blot was incubated with Notch antibody (top row) and the other was incubated with CBP antibody (3^rd^ row). Both blots were cut and incubated separately with tubulin antibody to serve as a control for protein loading and transfer (2^nd^ and 4^th^ rows). (C–D) are confocal images of dissected brains from control (no *Gal4*, *UAS-Ni*) and Notch-silenced experimental animals (*hsGal4; UAS-Ni*). Both groups were heat-shocked using the same regimen as B, and both were stained with CBP antibody and imaged using the same conditions and microscope settings.

To test this hypothesis, we first examined the genomic DNA surrounding the *CBP* transcription unit and its flanking regions for Su(H)/CBF1/RBP-Jκ (a transcription factor co-activated by Notch) binding sites. We identified four sites that matched the reported consensus sequences [26]. These occurred upstream and downstream (within the first intron) relative to the start of transcription (Fig. 2A), and the presence of these elements strengthened the model that *CBP* is transcriptionally regulated by the Notch ICD.

To test this possibility further, we analyzed CBP protein levels in adult flies in which *Notch* had been silenced with RNAi. The western analysis in Fig. 2B shows that when *Notch* is compromised (*hsGal4; UAS-Ni*, + heat shocks), CBP protein levels are similar to those detected in the controls [(no Gal4; *UAS-Ni*; + heat shocks) and (*hsGal4; UAS-Ni*; no heat shocks)]. Similar levels of CBP protein were also observed when control and *Ni* adult brains were compared using immunohistochemistry (Fig. 2C,D). We further analyzed the effects of Notch on *CBP* expression using qRT-PCR to measure *CBP* RNA levels from animals in which we either silenced *Notch* (with *UAS-Ni*) or overexpressed it (with *hsN+*). The results indicated that there was not a significant difference in *CBP* transcripts when these samples were compared to normalized wildtype levels (data not shown).

Taken together, these results suggest that the impaired LTM phenotype associated with *Notch* loss-of-function in the adult central nervous system cannot be easily explained by an appreciable effect on the expression of the *CBP* gene.

### Presenilin is Necessary for CBP Expression

Although we failed to establish a link between Notch and the expression of *CBP*, there are reports in the literature describing a connection between Psn and CBP [13], [14]. Thus, we used this transgenic RNAi strategy to ascertain whether CBP would be affected when *Psn* was silenced in adult flies.

**Figure 3:**
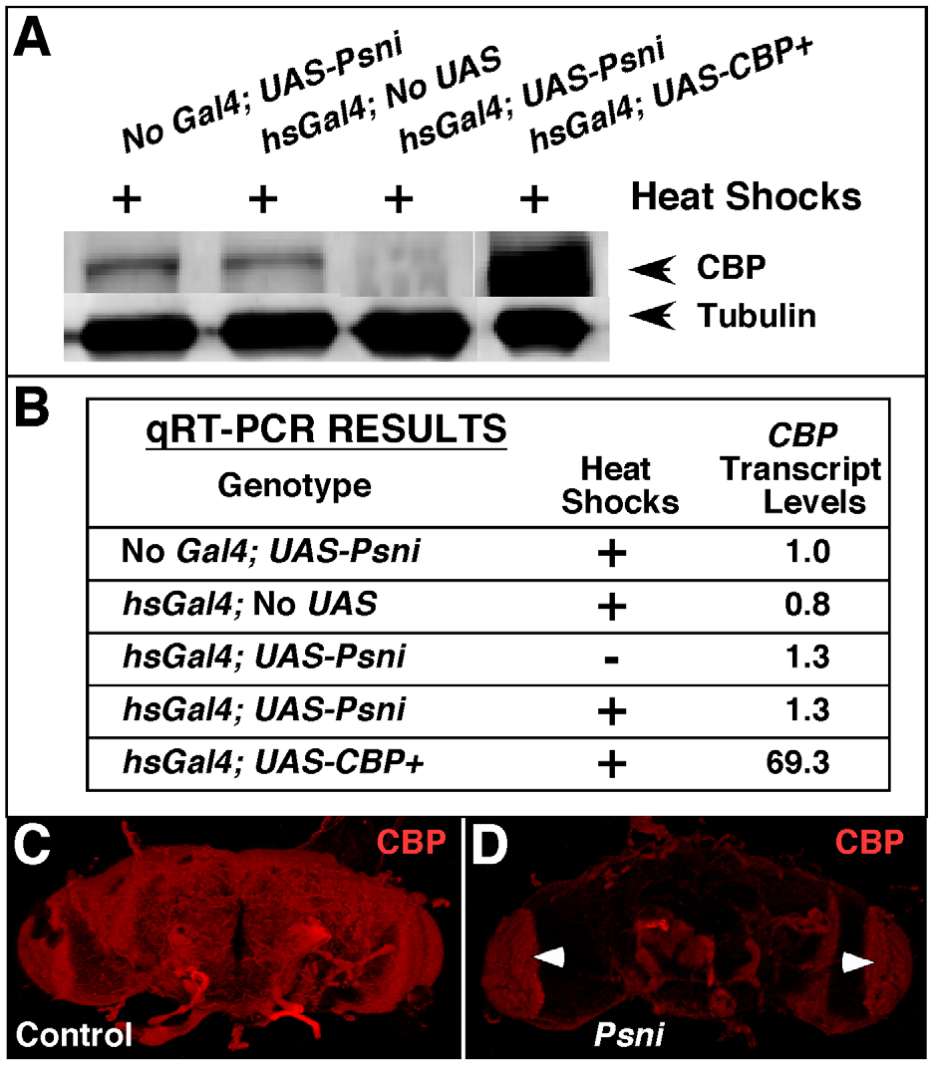
Psn is necessary for maintaining proper CBP protein levels in the adult *Drosophila* CNS. (A) shows a western analysis from fly heads in which CBP protein levels are reduced >90% (*hsGal4; UAS-Psni*; + heat shocks) compared to the controls (heat shocked animals without a driver or an RNAi responder) using tubulin as an internal standard for protein loading and transfer. (B) is a table of qRT-PCR results assaying *CBP* transcript levels (normalized relative to control  =  no *Gal4*; *UAS-Psni*) under conditions where *Psn* is silenced (*hsGal4; UAS-Psni*). An antibody stain on dissected heads indicates that CBP levels are drastically reduced when *Psn* is silenced (D) compared to a control without a driver (C). Arrowheads in (D) indicate non-specific autofluorescence of red pigment remaining after dissection.

As shown in the western-blot analysis of Fig. 3A, there is a ∼90% reduction in CBP protein in the Psn-reduced lane (*hsGal4; UAS-Psni; +* heat shocks) compared to the control (no *Gal4*; *UAS-Psni*; + heat shocks). However as demonstrated by the qRT-PCR experiment in Fig. 3B, *CBP* transcript levels are not significantly reduced when *Psn* is silenced with RNAi. Finally, in order to investigate whether Psn affects CBP accumulation in specific regions of the adult central nervous system, we performed antibody staining on dissected brains. Shown are representative examples of confocal Z-stack images of whole mounts from control [*no Gal4; UAS-Psni*; + heat shocks (Fig. 3C)] and experimental [*hsGal4; UAS-Psni*; + heat shocks (Fig. 3D)] groups. Clearly, there is a dramatic reduction in the amount of CBP protein detected when *Psn* is silenced. These data suggest that CBP is regulated by Psn in *Drosophila*, and given that Psn functions as a membrane-bound catalytic subunit of the γ-secretase, the mechanism probably requires intermediate signaling molecules that affect CBP protein accumulation and stability and is not a direct effect of transcriptional regulation.

### Global Acetylation Levels of H4K8 are Reduced in the Brain when the HAT Domain of CBP is Disrupted

Because the previous data suggests that Psn is required for *CBP* expression, we expect that phenotypes associated with mutations in *Psn* should overlap with mutations in *CBP*. One function of CBP that is critical to an active cell is the histone acetyl-transferase (HAT) function that modifies nucleosomes, presumably so that they can be more easily displaced by the transcription machinery. Therefore, the HAT activity of CBP could influence the acetylation of a few nucleosomes at key gene targets such as those that contain CREB response elements (CREs) for CREB-regulated transcription [27], or it might have global effects on chromatin structure throughout the genome [28].

**Figure 4:**
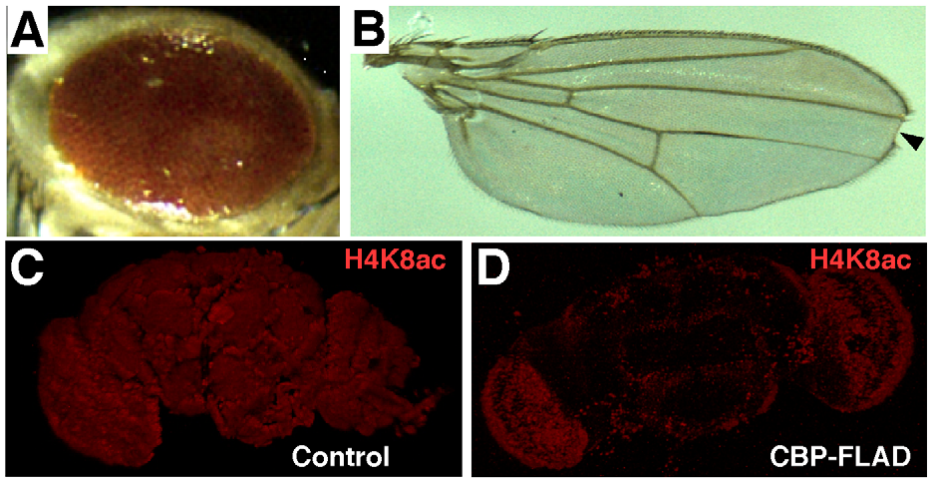
The HAT domain of CBP is required for H4K8ac in the adult *Drosophila* CNS. (A) depicts a typical roughened eye phenotype produced when *UAS-CBP-FLAD* is expressed with the *GMR-Gal4* driver during development. (B) displays a wing phenotype with a prominent notch (arrowhead) that is typically observed when *UAS-CBP-FLAD* is expressed with the *c96-Gal4* driver. Both phenotypes are similar to those detected when *UAS-CBPi* is expressed with the same drivers (Fig. 1). (C) is a control brain (no *Gal4*; *UAS-CBP-FLAD*; + heat shocks) stained for H4K8ac, and (D) is an experimental brain (*hsGal4; UAS-CBP-FLAD*; + heat shocks) stained with the same antibody under the same conditions. Note the dramatic reduction in staining intensity when the HAT-defective form of CBP is expressed.

To distinguish between these two possibilities, we used a dominant-negative variant of CBP in which the HAT domain is disrupted (*UAS-CBP-FLAD*) (see [29] for a complete description). When expressed in developing tissues, *UAS-CBP-FLAD* produces a roughened eye (Fig. 4A) and a notched wing (Fig. 4B, arrowhead), similar to the phenotypes observed when *UAS-CBPi* is expressed in the same tissues (Fig. 1D,L). We next compared whole-mount brains of control and *UAS-CBP-FLAD* adults for global acetylation levels by staining them with a well-characterized antibody that specifically recognizes acetylated lysines (at position 8) of histone 4 (H4K8ac). As shown, a dramatic reduction in H4K8ac staining was observed when control brains (Fig. 4C) were compared to those expressing the *UAS-CBP-FLAD* construct (Fig. 4D).

### Presenilin-Compromised Brains Also Exhibit Reductions in H4K8ac

**Figure 5:**
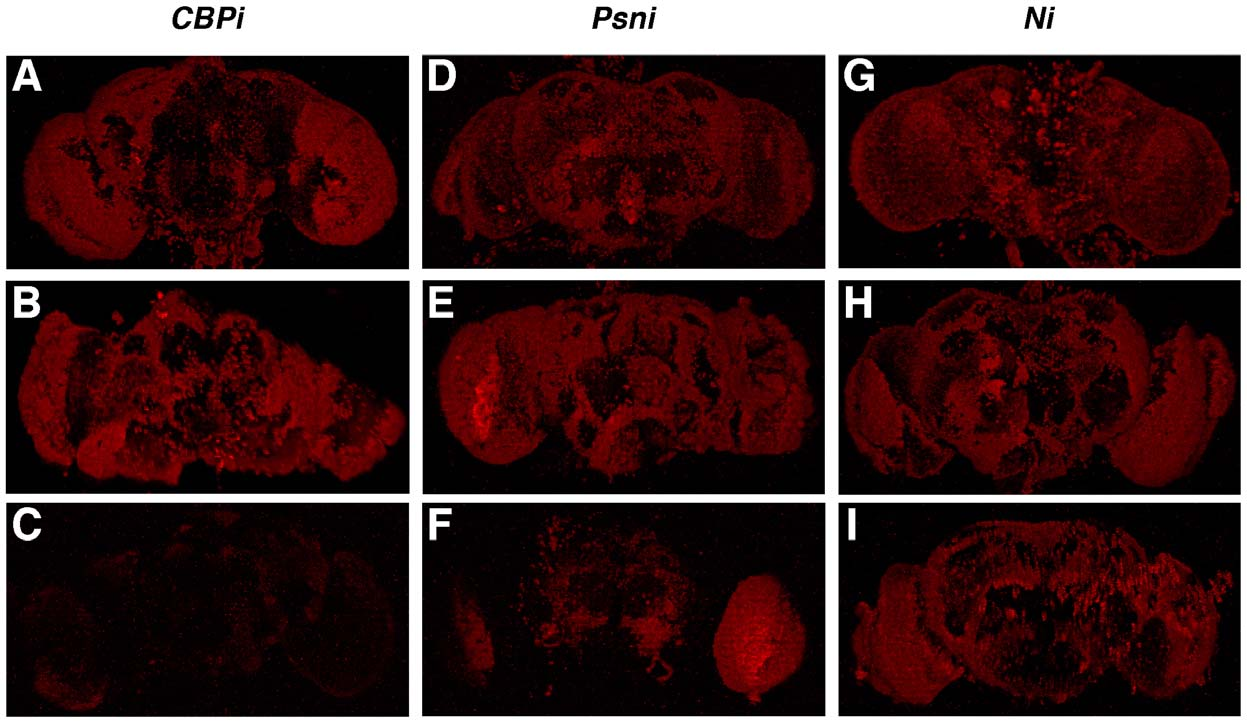
Psn affects H4K8ac levels in the adult brain. The above images are whole-mount adult fly brains stained for H4K8ac as an indicator of global acetylation levels. (A,D,G) represent one control group (*hsGal4; UAS-RNAi;* no heat shocks), (B,E,H) depict a second control group (no *Gal4*; *UAS-RNAi*; + heat shocks), and (C,F,I) represent the experimental group (hsGal4; *UAS-RNAi; +* heat shocks*).* Note that when *CBP* is silenced there is a dramatic effect on H4K8ac levels (compare C with the A and B controls); when *Psn* is silenced there is a significant but less dramatic effect (F); and when *Notch* is silenced (I) there is no noticeable effect. All brains were dissected, stained, and photographed using the same conditions and microscope settings.

To investigate whether Psn affects the overall levels of H4K8ac in the adult brain (as expected if Psn affects CBP function), we compared the staining patterns of dissected brains in which CBP (Fig. 5A-C), Psn (Fig. 5D-F), and Notch (Fig. 5G-I) had been silenced with RNAi. For each gene, two control groups were analyzed [(*hsGal4; UAS-RNAi*; no heat shocks) (Fig. 5A,D,G)] and [(no Driver; *UAS-RNAi*; + heat shocks) (Fig. 5B,E,H)]. It is clear from the data presented that H4K8ac levels are drastically reduced in brains expressing *UAS-CBPi* (Fig. 5C), moderately reduced in brains expressing *UAS-Psni* (Fig. 5F), and unaffected in brains expressing *UAS-Ni* (Fig. 5I).

These results demonstrate that CBP is needed for global acetylation at H4K8ac in the adult brain, and they are consistent with our model that Psn controls the accumulation/stability of CBP. They also suggest that reductions in Notch have little or no effect on H4K8ac levels within the limitations of our assays.

### CBP-Compromised Flies Exhibit an Altered Geotactic Response

Having demonstrated that CBP is needed for global levels of H4K8ac in the adult brain, we next wanted to investigate what effect, if any, these reductions had on the biology of the fly. In outward appearance, *Drosophila* compromised for CBP functions post development, appear normal. Both males and females are fertile and their lifespan is not dramatically affected. Therefore, in an effort to ascertain if they display any subtle behavioral phenotypes that might underlie neurological defects, we tested them for a normal geotactic response.

Some of the earliest discoveries that revealed a genetic basis for complex behavior were made by analyzing geotaxis—the movement of an organism in response to gravity [30], [31]. *Drosophila* exhibit strong negative geotaxis behavior because when placed in a tube, they prefer to climb toward the top. In addition, mutant studies have identified several genes that are required for this complex behavior [32].

**Figure 6:**
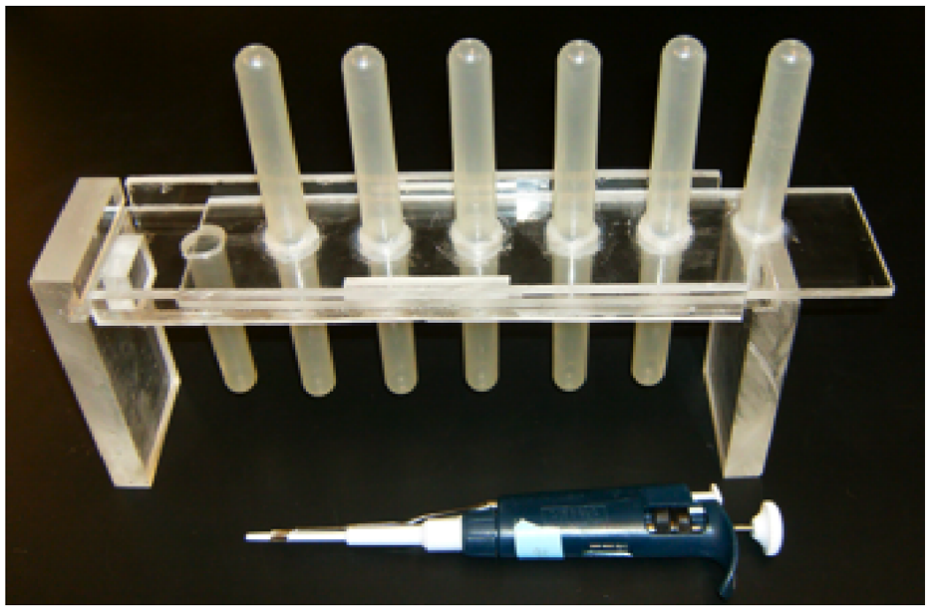
Assaying geotaxis using a countercurrent device. Pictured is the countercurrent device we built for assaying geotaxis behavior. This apparatus was modeled directly from a similar design created by Seymour Benzer [30]. Each experiment starts as flies are placed in tube set 1 and given 3 sharp taps on a rubber mat to knock them to the bottom. Flies are then given 10 seconds to climb into the upper tube, after which time it is moved over to the bottom of set 2. They are again tapped to the bottom, allowed to climb for 10 seconds, and moved to the bottom of set 3. The procedure continues until set 6 is reached. At the end of the experiment, the percentage of the fly cohort in each tube is calculated. The experiment is calibrated in such a way that most of the wildtype flies (exhibiting normal negative geotaxis) accumulate in tube number six.

Thus, to investigate whether CBP-compromised flies display an altered geotaxis, we built a countercurrent apparatus using specifications originally described by Seymour Benzer [30]. This apparatus monitors progressive geotactic performance as described in Fig. 6.

**Figure 7:**
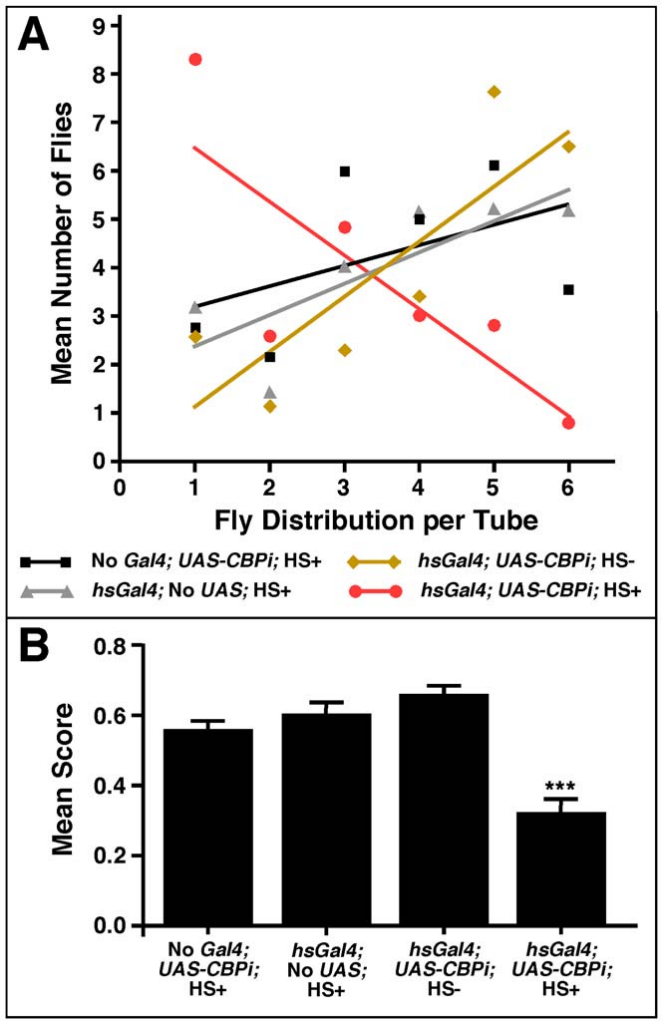
Flies compromised for *CBP* exhibit poor geotaxis. (A) represents the mean distribution of flies within each tube after 6 successive trials. Tube distribution for each group is presented by a best-fit line, which is based on a linear regression. The slope of the experimental group (*hs-Gal4; UAS-CBPi*) indicates that the majority of these cohorts remained in the first sets of tubes, whereas the control groups accumulated in the last sets of tubes. (B) is the mean score for each group with a significant difference between the experimental group (*hsGal4*; *UAS-CBPi;* +heat shocks) and all three control groups (*F*_(3,53)_ = 11.8; *P*<0.0001). There were no significant differences between genders or control groups. A (+) indicates a heat shock treatment and (-) indicates no heat shock.

The mean tube distribution for four different cohorts of flies—three controls (without driver, without responder, without heat shocks) and one experimental—are presented in Fig. 7A. Clearly most of the flies in the experimental group (*hsGal4; UAS-CBPi; +*heat shocks) were impaired in their ability to progress beyond the first tube. In addition, an ANOVA analysis of the data indicates that the performance of the experimental group is significantly impaired (*F*_(3,53)_ = 21.02; *P*<0.0001) compared to the performance of each control group (Fig. 7B). These results suggest that CBP is required in the adult animal for the maintenance of some normal behaviors, and the fact that CBP-compromised flies display this behavioral phenotype may be indicative of some type of neurological defect associated with its dysfunction.

## Discussion

Based on the data presented in this report, we have provided clear evidence to support the conclusion that CBP is required to maintain global levels of H4K8 acetylation in the adult *Drosophila* CNS. We base our conclusions on the powerful combination of using both loss-of-function (expression of RNAi against CBP) and dominant-negative (expression of CBP-FLAD) genetic reagents (Fig. 4D; Fig. 5C). We also show that when CBP expression is uncoupled from development and conditionally silenced only in adults, we detect subtle changes in the behavior of the fly that can be easily quantified in a geotaxis assay (Fig. 7B). Because CBP is a multifunctional protein that co-activates so many genes and because CBP deficiencies have drastic developmental effects, our results are surprising in the sense that the phenotype described in these adult flies is not more severe. There are at least two possible explanations for this observation.

The first concerns the limitations of the inducible RNAi strategy, and the fact that after multiple rounds of heat shocks *CBP* is not completely silenced (data not shown). Consequently, the remaining low levels of CBP may supply enough vital functions for the fly. The second possibility speaks to the function of CBP in acetylating histones, and the fact that we monitored H4K8ac levels. In this model, the adult nervous system is supplied with wildtype levels of CBP during development, but as levels drop during our experimental manipulations of aging adults, histone modifications required for more open chromatin configurations are slowly eroded. Thus, subtle nervous system activities that control behavioral functions, such as negative geotaxis, would likely be affected first, followed by more vital functions. Based on our observations with *Notch* loss-of-function manipulations in adults [12], we favor the latter explanation, and we are currently testing this model further by examining more complex behaviors such as learning and memory under conditions in which *Psn* and *CBP* are compromised in aging animals. Finally, if this model is correct, understanding the role of CBP (and molecules that affect its activity such as Psn) in an aging nervous system might have important implications for the study of human neurodegenerative diseases. For example, subtle genetic mutations in *CBP* may be amplified if a person has a specific environmental exposure to an agent that modifies chromatin structure.

A second significant conclusion from this report is that we clearly establish a relationship between Psn and CBP protein accumulation in the fly. We show that when *Psn* is silenced with RNAi, we see dramatically reduced levels of CBP Protein (Fig. 3A and C) and similar effects on global acetylation levels of H4K8ac (Fig. 5F). In addition, our results are interesting because reports examining the link between Psn and CBP in mice are contradictory. One group reported that the Psn-processed N-cadherin ICD targets CBP for degradation, and that Psn FAD-linked mutant mouse embryonic fibroblasts showed increased levels of CBP protein [14]. However, another reported that conditional knockdowns of both *Psn* homologues (*PS1/PS2*) showed a reduction in CBP protein and mRNA levels in the mouse hippocampus [13]. Our results are similar to the latter study in that CBP protein levels are reduced in a Psn-reduced background, however we did not observe the subtle reduction in *CBP* transcripts (∼20%) that they reported (Fig. 3B). Perhaps the fly system can be used in combination with the mouse system to clear up the discrepancy and/or speak to the divergent pathways that can explain these results in vertebrates. In either case the relationship between Psn activity and *CBP* expression is probably not direct given our understanding of Psn as a transmembrane protein that processes type I receptors.

We and others have clearly demonstrated that when Notch is compromised in the nervous system of adult animals (flies or mice) LTM is specifically impaired [12], [21], [22]. Initially, we began this analysis with the expectation that Notch, Psn, and CBP would be linked in a common pathway in which a Psn-processed form of Notch would co-activate target genes that regulate global acetylation levels. The model was attractive because it had the potential to connect a molecular “memory” of histone acetylation and placement with an organic explanation of how an organism's long-term memory is either established or maintained. We were encouraged to pursue this model when we were able to identify four putative Su(H) binding sites within 2.1 kB of the start of transcription of *CBP* (Fig. 2A), but we were unable to show that compromising *Notch* had any effect on *CBP* RNA levels, CBP protein levels, or H4K8 acetylations (Fig. 2B, Fig. 5I). Also, mouse brains compromised for Psn show reduced CREB activity, where early hypotheses suggested that Psn influences CRE-regulated genes through Notch signaling-induced CBP coactivation [13]. However, a recent report shows that Notch signaling does not directly affect CREB activity [33]. Nevertheless it still remains a formal possibility that in key neurons of the fly brain that control LTM, a large reduction in Notch could have a subtle reduction in CBP, and that this subtle reduction is responsible for the LTM phenotype that is observed. Alternatively, it is possible that one of the functions of CBP is influenced by Notch in a manner which may go undetected using our techniques.

We are still unsure how Psn and CBP are connected at the molecular level. One possibility is that the Psn processing of another type I receptor leads to a transcriptional control of *CBP*. With the availability of inducible RNAi constructs for most of the known *Drosophila* Psn substrates [34] this possibility could now be tested. A second possibility is that the activity of some of the protein targets that interact non-catalytically with Psn may become altered when Psn is reduced, and their dysfunction might affect CBP expression or stability. However, a third possibility exists that Psn regulates CBP through a mechanism that is currently unknown.

Regardless of mechanism, our data clearly link Psn and CBP in a pathway that is required for normal H4K8ac levels and for normal behavioral activities. In the vertebrate literature, Psn is linked to AD, and CBP is associated with specific mental disorders including Rubenstein-Taybi and Huntington's disease.

The rapidly increasing list of Psn substrates and associating proteins not only suggests a broader role for Psn, but also reveals new therapeutic targets in AD research. The use of histone deacetylase (HDAC) inhibitors may prove to be useful as a treatment designed to arrest the neurodegeneration associated with AD, but the cause for these certain forms of AD may reside in the increasingly complex networks integrated through Psn, and therefore more research is needed to elucidate their functions.

## Materials and Methods

### Drosophila Stocks and Culture

All flies were raised on standard cornmeal-molasses medium supplemented with live baker's yeast using the recipe recommended by the Bloomington Stock Center (Bloomington, Indiana, United States) (http://flystocks.bio.indiana.edu/Fly_Work/media-recipes/bloomfood.htm).

The stocks listed below were obtained from Bloomington: *w^1118^* (FBst0307124), *hs-CBP+* (FBst0003730), UAS-*Psn+* (FBst0008309), *UAS-Psni* [selected as described below (FBst0008317)], *GMR-Gal4* (FBst0001104), *pnr-Gal4* (FBst0003039), and *UAS-Ni* (FBst0007078).

The following stocks were obtained as gifts: *hs-N+* and *c96-Gal4* [35] from S. Artavanis-Tsakonas; *UAS-CBP+* and *UAS-CBPi* [20] from J. Kumar; *UAS-CBP-FLAD* [29] from S. Smolik; and *hsGal4* [15] from R. Holmgren.

### Generation of Transgenic UAS-Psni Fly Lines

Two separate DNA fragments of 262 base pairs were amplified from the ninth exon of the *Drosophila Psn* gene (Fig. 2A) using two separate primer sets (Integrated DNA Technologies, Coralville, Iowa, United States). The first primer set (GGGAATTCGGCATAAAGCTTGGCCTC and GGCTCGAGTATAAACACCTGCTTGGC) produced a fragment that was digested with *EcoRI* and *XhoI* and directionally cloned into the corresponding sites of the *pWIZ* vector downstream of *UAS* sequences [36]. The second primer set (GGGCTAGCTATAAACACCTGCTTGGC and GGTCTAGAGGCATAAAGCTTGGCCTC) amplified the same piece of DNA. However, it was digested with *NheI* and *XbaI* and directionally cloned into *pWIZ* to produce an inverted repeat of the first fragment. The completed *pWIZ-Psni* construct was sent to the Holmgren Lab (Northwestern University, Evanston, Illinois, United States) for the production of transgenic flies using standard techniques. Eight transgenic lines were produced: *UAS-Psni-J1, UAS-Psni-J2, UAS-Psni-J3, UAS-Psni-J5, UAS-Psni-J7, UAS-Psni-2a, UAS-Psni-2b,* and *UAS-Psni-15*.

### Selecting an effective UAS-Psni Line

During the period when we were generating *UAS-Psni* transgenic flies, four independently produced lines became available from the *Drosophila* community. All of these contained inverted repeats of *Psn* sequences under *UAS* control. Three lines came from Bloomington (FBti0040721, FBti0040747, FBti0040722) and one line from the Vienna *Drosophila* RNAi Center (*UAS-Psni-43082*) [34].

We tested all 12 lines by expressing them in the developing wing, thorax, and eye (Fig. 1C,G,K) and selected the line that produced the most severe phenotypes.

### Western Blotting

Protein extracts of adult *Drosophila* heads were prepared as described in [27]. They were removed from anesthetized flies of the appropriate genotype with a razor blade and homogenized in a lysis buffer (1% NP-40, 0.5% deoxycholic acid, 0.1% Triton-X-100, 100 mM NaCl, 0.1 mM CaCl_2_, 2 mM MgCl_2_) supplemented with freshly prepared protease inhibitor cocktail (Sigma-Aldrich Inc., St. Louis, Missouri, United States) to a final concentration of 1%. Cell debris was pelleted in a microfuge (1000×g for 1 minute) and the supernatant was transferred to an equal volume of 2× Laemmli buffer (2x = 4% SDS, 20% glycerol, 120 mM Tris pH 6.8, 10% β-mercaptoethanol, 0.002% bromophenol blue). The equivalent of 7.5 heads for Notch detection and 2.5 heads for CBP detection were boiled for 5 minutes and loaded on a 7.5% polyacrylamide gel.

Gels were blotted and treated with antibodies using a modified protocol [37]. After electrophoresis, the gel was electroblotted onto Immobilon-P membrane (Millipore, Bedford, Massachusetts, United States) for 2.5 hours and blocked overnight in PBST (1.4 M NaCl, 26.8 mM KCl, 101.4 mM Na_2_PO_4_, 17.6 mM KH_2_PO_4_, and 1% Tween 20) plus 5% dry milk (Nestle USA Inc., Solon, Ohio, United States) at 4°C. The following primary antibodies were used: rabbit polyclonal anti-dCBP (a gift from Alexander Mazo) diluted 1∶1000, mouse monoclonal anti-Notch C17.9c6 (Developmental Studies Hybridoma Bank at the University of Iowa, Iowa City, Iowa, United States) diluted 1∶2000, and mouse monoclonal anti-α-tubulin (Sigma-Aldrich) diluted 1∶15,000. Primary antibody incubation was done in PBST and 5% milk overnight at 4°C. Goat-anti-mouse-HRP diluted 1∶25,000 and Goat-anti-rabbit-HRP diluted at 1∶40,000 (Jackson Immuno Research, West Grove, Pennsylvania, United States) were used as secondary antibodies. An ECL Plus Detection System (GE Healthcare, Piscataway, New Jersey, United States) was used for protein detection and visualized on a Typhoon 8600 Variable Mode Phosphorimager (GE Healthcare).

### Immunohistochemistry

Flies of the appropriate genotype were selected from progeny that were raised at 18°C and collected 3–5 days post eclosion. They were subjected to a heat shock regimen to induce the proper transgene by either being moved to 29°C for the duration of the experiment or by being immersed in their culture tubes in a 37°C water bath as described below. Brains were dissected in PBS (1.9 mM NaH_2_PO_4_, 8.4 mM Na_2_HPO_4_, 175 mM NaCl), fixed in 4% paraformaldehyde (in PBS), and treated with primary and secondary antibody [38]. The following primary antibodies were used: rabbit polyclonal anti-CBP (Alexander Mazo) diluted 1∶350 in PBSBT (PBS, 0.2% Triton-X100, 0.25% BSA) and rabbit polyclonal anti-H4K8ac (Santa Cruz Biotechnology, Inc., Santa Cruz, California, United States) diluted 1∶350 in PBSBT. Incubation in primary antibody occurred overnight with gentle rocking at 4°C. After washing 3×5 minutes in PBT (PBS, 0.2% Triton-X100) the brains were incubated with Rhodamine-conjugated goat-anti-rabbit secondary antibody (Jackson Immuno Research), diluted 1∶1000 in PBSBT for 4 hours at room temperature. The final washes (3×5 minutes followed by 3×20 minutes) were done in PBT. The tissues were mounted in Flouro-Gel (Electron Microscopy Sciences, Hattfield, Pennsylvania, United States) and imaged on a LSM 510 Axioplan confocal microscope (Carl Zeiss SMT, Peabody, Massachusetts, United States) equipped with LSM 510 image-analysis software (Carl Zeiss).

### Quantative Real-Time (qRT) PCR

RNA was extracted from 100 adult *Drosophila* heads using the RNeasy Mini Kit (Qiagen, Valencia, California, United States) and treated with Turbo DNA-*free* (Ambion Inc., Austin, Texas, United States) using their respective protocols. cDNAs were synthesized using SuperScript First-Strand (Invitrogen, Carlsbad, California, United States) according to manufacturer's instructions. GAGCAGGCGGTAATCTTCAG and TTGCTGGGGAAGAACTATGG primers (Integrated DNA Technologies) and PerfeCTa SYBR Green SuperMix (Quanta Biosciences, Gaithersburg, Maryland, United States) were used to amplify and detect *CBP* sequences during the reactions. Each reaction was performed in triplicate on a Bio-Rad iCycler iQ Real Time PCRSys system (Bio-Rad Laboratories, Hercules, California, United States), and experimental ct values were normalized to *Beta-Actin* using TCTACGAGGGTTATGCCCTT and GCACAGCTTCTCCTTGATGT primers. The data was subjected to ΔΔCt statistical analysis.

### Heat Shock Treatments

For experiments that involved the conditional induction of transgenes, flies were raised at 18°C until 3–5 days post-eclosion. They were then either moved to 29°C (for experiments using *UAS-CBP-FLAD*) or subjected to heat shocks in a 37°C waterbath (for experiments involving *UAS-Ni, UAS-Psni*, *UAS-CBPi, UAS-N+, UAS-Psn+,* or *UAS-CBP+*). Prior to administering the heat shocks, flies were transferred to 9.5×2.5 cm polypropylene vials containing 8 ml of cornmeal-molasses medium covered with a thin layer of rayon to protect them from dehydration and getting stuck in the food. Buzz plugs (Genesee Scientific, San Diego, California, United States) were used to create a chamber that was submerged in the water bath. Conditions for multiple heat shocks were determined empirically for each genotype to maximize target-gene silencing (determined by western blot) without causing lethality.

The following heat shock regimens were used: 7 one-hour treatments (∼12–14 hours apart) for *CBPi (hsGal4; UAS-CBPi)* and controls; 9 one-hour treatments (∼12–14 hours apart) for *Ni (hsGal4; UAS-Ni), Psni (hsGal4; UAS-Psni)*, and their control groups; and 2 one-hour treatments (∼12–14 hours apart) for *N+ (hsN+), CBP+ (hsCBP+* and *hsGal4; UAS-CBP+)*, and controls.

Flies recovered between heat shocks at 25°C and were given a one-hour rest period at 25°C after the last treatment before their brains were dissected.

### Geotaxis Response Assay

All geotaxis experiments were performed in a countercurrent apparatus as previously described [30]. The apparatus consists of 6 sets of polypropylene tubes (9×1.5 cm) vertically opposed. The opposing tubes are held in a plexiglass frame that allows the tubes to move in unison relative to each other (Fig. 6). The experiments were conducted without light in an environmentally controlled chamber at 25°C with 65% relative humidity, and all fly lines were outcrossed for 7 generations to reduce the occurrence of modifiers.

Roughly 25 flies of mixed sex were used per round. Flies were given a score based on how far they advanced by the conclusion of the round. The percentage of the cohort in tube 6 was multiplied by 1, those in tube 5 by 0.8, those in tube 4 by 0.6, those in tube 3 by 0.4, those in tube 2 by 0.2 and those in tube 1 by 0. These scores are totaled for each round and the mean and standard error were calculated based on a total of twelve different rounds. All groups were compared for significance using a one-way ANOVA using GraphPad Prism software (La Jolla, California, United States).

### Accession Numbers

The FlyBase (http://flybase.bio.indiana.edu/search/) identification numbers are used in this work to describe *Drosophila* stocks.

## References

[1] Tanzi RE, Bertram L (2005). Twenty years of the Alzheimer's disease amyloid hypothesis: A genetic perspective.. Cell.

[2] Beel AJ, Sanders CR (2008). Substrate specificity of gamma-secretase and other intramembrane proteases.. Cellular and molecular life sciences.

[3] Parks AL, Curtis D (2007). Presenilin diversifies its portfolio.. Trends in Genetics.

[4] Hass MR, Sato C, Kopan R, Zhao G (2009). Presenilin: RIP and beyond.. Seminars in cell & developmental biology.

[5] Shen J, Kelleher RJ (2007). The presenilin hypothesis of Alzheimer's disease: Evidence for a loss-of-function pathogenic mechanism.. Proceedings of the National Academy of Sciences.

[6] Wines-Samuelson M, Shen J (2005). Presenilins in the developing, adult, and aging cerebral cortex.. The Neuroscientist.

[7] Kasper LH, Fukuyama T, Biesen MA, Boussouar F, Tong C (2006). Conditional knockout mice reveal distinct functions for the global transcriptional coactivators CBP and p300 in T-cell development.. Molecular and cellular biology.

[8] Barrios-Rodiles M, Brown KR, Ozdamar B, Bose R, Liu Z (2005). High-throughput mapping of a dynamic signaling network in mammalian cells.. Science's STKE.

[9] Goodman RH, Smolik S (2000). CBP/p300 in cell growth, transformation, and development.. Genes & development.

[10] McCampbell A, Taylor JP, Taye AA, Robitschek J, Li M (2000). CREB-binding protein sequestration by expanded polyglutamine.. Human molecular genetics.

[11] Rubenstein JH, Taybi H (1963). Broad thumbs and toes and facial abnormalities: a possible mental retardation syndrome.. Archives of Pediatrics & Adolescent Medicine.

[12] Presente A, Boyles RS, Serway CN, De Belle JS, Andres AJ (2004). Notch is required for long-term memory in *Drosophila*.. Proceedings of the National Academy of Sciences.

[13] Saura CA, Choi SY, Beglopoulos V, Malkani S, Zhang D (2004). Loss of presenilin function causes impairments of memory and synaptic plasticity followed by age-dependent neurodegeneration.. Neuron.

[14] Marambaud P, Wen PH, Dutt A, Shioi J, Takashima A (2003). A CBP binding transcriptional repressor produced by the PS1/-cleavage of N-cadherin is inhibited by PS1 FAD mutations.. Cell.

[15] Brand AH, Perrimon N (1993). Targeted gene expression as a means of altering cell fates and generating dominant phenotypes.. Development.

[16] Presente A, Shaw S, Nye JS, Andres AJ (2002). Transgene-mediated RNA interference defines a novel role for notch in chemosensory startle behavior.. Genesis.

[17] De Celis JF, Garcia-Bellido A, Bray SJ (1996). Activation and function of Notch at the dorsal-ventral boundary of the wing imaginal disc.. Development.

[18] Heitzler P, Simpson P (1993). Altered epidermal growth factor-like sequences provide evidence for a role of Notch as a receptor in cell fate decisions.. Development.

[19] Baker NE, Yu SY (1998). The R8-photoreceptor equivalence group in Drosophila: fate choice precedes regulated Delta transcription and is independent of Notch gene dose.. Mechanisms of Development.

[20] Kumar JP, Jamal T, Doetsch A, Turner FR, Duffy JB (2004). CREB binding protein functions during successive stages of eye development in *Drosophila*.. Genetics.

[21] Ge X, Hannan F, Xie Z, Feng C, Tully T (2004). Notch signaling in *Drosophila* long-term memory formation.. Proceedings of the National Academy of Sciences of the United States of America.

[22] Costa RM, Honjo T, Silva AJ (2003). Learning and memory deficits in Notch mutant mice.. Current Biology.

[23] Bourtchuladze R, Frenguelli B, Blendy J, Cioffi D, Schutz G (1994). Deficient long-term memory in mice with a targeted mutation of the cAMP-responsive element-binding protein.. Cell.

[24] Mantamadiotis T, Lemberger T, Bleckmann SC, Kern H, Kretz O (2002). Disruption of CREB function in brain leads to neurodegeneration.. Nature genetics.

[25] Lonze BE, Ginty DD (2002). Function and regulation of CREB family transcription factors in the nervous system.. Neuron.

[26] Nellesen DT, Lai EC, Posakony JW (1999). Discrete enhancer elements mediate selective responsiveness of enhancer of split complex genes to common transcriptional activators.. Developmental biology.

[27] Vecsey CG, Hawk JD, Lattal KM, Stein JM, Fabian SA (2007). Histone deacetylase inhibitors enhance memory and synaptic plasticity via CREB: CBP-dependent transcriptional activation.. Journal of Neuroscience.

[28] Cheng CS, Johnson TL, Hoffmann A (2008). Epigenetic control: slow and global, nimble and local.. Genes & development.

[29] Ludlam WH, Taylor MH, Tanner KG, Denu JM, Goodman RH (2002). The acetyltransferase activity of CBP is required for wingless activation and H4 acetylation in *Drosophila melanogaster*.. Molecular and cellular biology.

[30] Benzer S (1967). Behavioral mutants of *Drosophila* isolated by countercurrent distribution.. Proceedings of the National Academy of Sciences of the United States of America.

[31] Erlenmeyer-Kimling L, Hirsch J (1961). Measurement of the relations between chromosomes and behavior.. Science.

[32] Toma DP, White KP, Hirsch J, Greenspan RJ (2002). Identification of genes involved in *Drosophila melanogaster* geotaxis, a complex behavioral trait.. Nature genetics.

[33] Watanabe H, Smith MJ, Heilig E, Beglopoulos V, Kelleher RJ (2009). Indirect regulation of presenilins in CREB-mediated transcription.. Journal of Biological Chemistry.

[34] Dietzl G, Chen D, Schnorrer F, Su KC, Barinova Y (2007). A genome-wide transgenic RNAi library for conditional gene inactivation in *Drosophila*.. Nature.

[35] Rebay I, Fehon RG, Artavanis-Tsakonas S (1993). Specific truncations of *Drosophila* Notch define dominant activated and dominant negative forms of the receptor.. Cell.

[36] Takemaru KI, Yamaguchi S, Lee YS, Zhang Y, Carthew RW (2003). Chibby, a nuclear beta-catenin-associated antagonist of the Wnt/Wingless pathway.. Nature.

[37] Vaskova M, Bentley AM, Marshall S, Reid P, Thummel CS (2000). Genetic analysis of the *Drosophila* 63F early puff: Characterization of mutations in E63-1 and maggie, a putative Tom22.. Genetics.

[38] Wulbeck C, Helfrich-Forster C (2007). RNA in situ hybridizations on *Drosophila* whole mounts.. Methods in Molecular Biology-Clifton Then Totowa-.

